# Laparoscopic nephrectomy for Xanthogranulomatous Pyelonephritis: First surgical outcomes report from Pakistan

**DOI:** 10.12669/pjms.42.(11AASC).15801

**Published:** 2026-04

**Authors:** Ali Anjum Fazal, Syed Muhammad Nazim, Imran Khan Jalbani, Zeeshan Uddin

**Affiliations:** 1 Dr. Ali Anjum Fazal, MBBS, Section of Urology, Department of Surgery, The Aga Khan University Hospital, Karachi, Pakistan; 2 Dr. Syed Muhammad Nazim, FCPS, FRCS, FACS, Section of Urology, Department of Surgery, The Aga Khan University Hospital, Karachi, Pakistan; 3 Dr. Imran Khan Jalbani, FCPS, Section of Urology, Department of Surgery, The Aga Khan University Hospital, Karachi, Pakistan; 4 Dr. Zeeshan Uddin, FCPS, FRCPath, Section of Urology, Department of Surgery, The Aga Khan University Hospital, Karachi, Pakistan

**Keywords:** Laparoscopic, LMIC, Minimally invasive surgery, Pakistan, Simple nephrectomy, Xanthogranulomatous pyelonephritis

## Abstract

**Objectives:**

The aim of this study was to analyze the perioperative and postoperative outcomes of laparoscopic nephrectomy (LN) in the management of patients with xanthogranulomatous pyelonephritis (XGPN).

**Methodology:**

This retrospective review examined medical records of patients who underwent LN for histopathologically confirmed XGPN at the Aga Khan University Hospital, Pakistan, from January 2019 to October 2024. Patient demographics, clinical presentation, microbiological findings, imaging results, operative details, and postoperative outcomes including hospital stay, complications, and 30-day readmission were evaluated.

**Results:**

A total of 13 LNs were performed for XGPN. The mean patient age was 49.6 ± 17.3 years, with females accounting for 61.5% of cases. Flank pain was the most common presenting symptom, while hypertension and diabetes mellitus were the most frequent comorbidities (23.1% each). Most preoperative urine cultures were negative; among positive cultures, *Escherichia coli* was the predominant pathogen. Urinary tract stones were present in 84.6% of patients. Preoperative percutaneous nephrostomy was performed in majority (61.5 %). Renal scans in all patients revealed markedly impaired renal function. LN was completed successfully in all cases without conversion to open surgery. The mean operative time was 194 + 49 minutes, with minimal blood loss and an average hospital stay of three days. Postoperative complications occurred in two patients (15.4%), and no patient required readmission within 30 days. Survival was 100% among those with at least one year of follow-up.

**Conclusion:**

Laparoscopic nephrectomy is a feasible, safe, and effective treatment option for XGPN in our setting and is associated with favorable surgical outcomes.

## INTRODUCTION

Xanthogranulomatous pyelonephritis (XGPN), a rare chronic inflammatory kidney disease that represents 0.6-1% of all kidney infections, poses unique surgical challenges.^1^ Nephrectomy for XGPN carries significantly higher morbidity due to dense fibrosis, distorted anatomical planes, and extensive adhesions.^1^ For this reason, many expert urologists argue that the term “simple nephrectomy” is a misnomer in XGPN and have proposed renaming it to “complex” or “chaotic” nephrectomy.^2^,^3^ In Pakistan and other low- and middle-income countries (LMICs), open nephrectomy remains more common because laparoscopic surgery requires specialized skills, advanced equipment, and higher upfront costs. However, laparoscopic nephrectomy may offer long-term economic benefits by reducing hospital stay, analgesic requirements, and time off work which are critical considerations.^4^,^5^ Despite these implications, evidence regarding the outcome of laparoscopic nephrectomy for XGPN remains scarce, particularly in LMICs.^6^ This study addresses this gap by evaluating surgical outcomes in patients undergoing laparoscopic nephrectomy for XGPN at a tertiary care center in Pakistan. This is the first study from our country and among the few from LMIC.

## METHODOLOGY

This retrospective cohort study was conducted at the Section of Urology- Aga Khan University Hospital, Karachi-Pakistan. Ethics review committee approval was obtained (ERC-2025-11790-35956; date July 31, 2025). The study included all eligible patients aged 18 years or more who underwent laparoscopic nephrectomy and had a histo-pathologically proven diagnosis of Xanthogranulomatous Pyelonephritis, with follow-up data up to one year, between January 2019 to October 2024. A total population sampling strategy was used in which all eligible patients meeting inclusion criteria within the defined time frame were included. Patients who were lost to follow-up or had insufficient data available were excluded from the study. We reviewed the records of all patients who underwent laparoscopic nephrectomy during the study period and identified those with a histopathological diagnosis of XGPN. Out of a total of 186 laparoscopic nephrectomies performed, 13 patients with confirmed XGPN comprised the final study cohort.

Data was recorded in RedCap using a pre-designed proforma. We recorded demographic data including age, gender, body mass index (BMI), comorbidities and presenting complaints. Relevant laboratory & Microbiological parameters including pre-operative urine culture were also recorded. All patients received antibiotic treatment according to the report of urine culture and sensitivity. Pre-operative CT scan and Renal scan (DMSA or MAG-3) was done in all patients to assess the disease process (stone burden/extent of inflammation); and split renal function of the affected kidney respectively. Any pre-operative intervention such as placement of Percutaneous nephrostomy tube (PCN) or JJ stent was also recorded.

All patients underwent a standardized three port (left-sided) or four port (right-sided) transperitoneal laparoscopic nephrectomy under general anesthesia using a 30° laparoscope. Port placement included a 12mm lateral optical trocar in the lower quadrant for the primary working port, a 10mm camera port placed just medial to the lateral rectus or near the midline, and one or two additional 5mm trocars in the upper quadrants for retraction and auxiliary instrumentation.

After creation of pneumoperitoneum, a standard transperitoneal laparoscopic nephrectomy was performed. Dissection began using LigaSure™ device and included colon mobilization, adhesiolysis, renal mobilization, and hilar dissection with vascular control. To prevent contamination or spillage into peritoneal cavity or wound site, the specimen was retrieved intact using a low-cost, modified endoscopic retrieval bag fashioned from a sterile urine collection bag introduced through the 12-mm optical port and extracted within the bag by extending the port incision in a muscle splitting fashion. All patients received standardized multimodal analgesia consisting of intravenous paracetamol 1g every six hours, with intravenous tramadol 50mg and metoclopramide as required for breakthrough pain or postoperative nausea.

The intra-operative findings such as operative time, estimated blood loss and perioperative parameters such as analgesia requirement, length of hospital stay and 30 days perioperative complications and readmission within 30 days and survival were assessed.

Data was analyzed using R studio. Both quantitative and qualitative variables were appropriately summarized: depending on the data distribution, continuous variables were given as means with standard deviations or medians with interquartile ranges, while categorical variables were stated as frequencies and percentages.

## RESULTS

The mean age of patients was 49.62 ± 17.3 years (range: 20–77 years), with the majority (n=8, 61.5%) being women. The mean BMI was 22.9 ± 4.4 kg/m² (range: 17.3–30). All 13 patients were diagnosed with unilateral XGPN, with laterality being left-sided in 7 (53.8%) and right-sided in 6 (46.2%) patients.

Flank pain was reported in all 13 cases, with five cases (38.5%) presenting with fever and Lower urinary tract symptoms (LUTS) & dysuria in 6 (46 %). One (7.7%) case reported nausea & vomiting. Diabetes Mellitus (DM) & Hypertension (HTN) were present in 3 cases (23.1%) each and 4 patients had chronic kidney disease (n=2) and ischemic heart disease (n=2). One patient each had, breast cancer, prostate cancer, and Hepatitis C. Mean pre-operative hemoglobin was 10.6 ± 1.67 gm/dl (range: 9.0-13.7) and mean C-reactive protein (CRP) was 85.09 ± 73.7 mg/l (range: 22.6-279). Mean pre-operative serum creatinine was 1.22 ± 0.45 mg/dl (range: 0.6-2.6) and mean serum creatinine at the time of discharge was 1.19 +/- 0.52 mg/dl (0.6-2.4).

**Fig.1:**
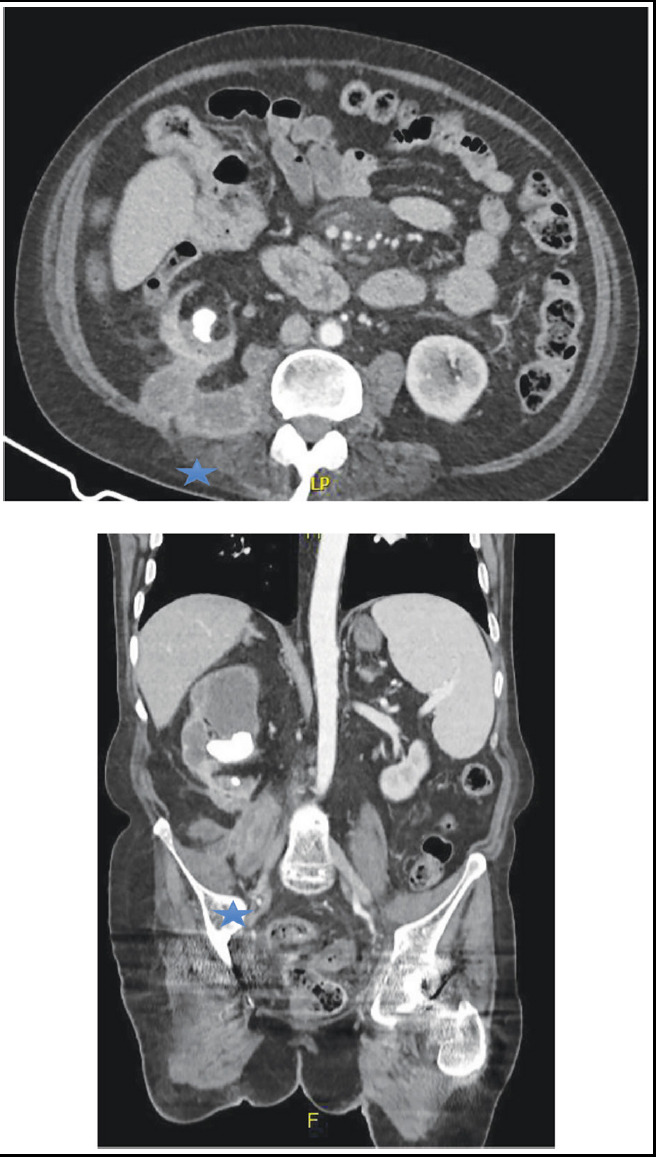
A & B Axial and coronal images of contrast enhanced CT abdomen showing grossly hydronephrotic right kidney with loss pf parenchyma and multiple stones in collecting system. Peri-nephric abscess is also appreciated (Blue star).

The majority of the preoperative urine culture showed no growth (n=8, 61.5%). In the remaining cultures, *Escherichia coli* was the most common pathogen, found in two of the cases (15.4%). Other pathogens found were *Proteus mirabilis* (n=1, 7.7%), *Pseudomonas aeruginosa* (n=1, 7.7%), and *Enterococcus* species (n=1, 7.7%). The presence of stones in the affected side of the urinary tract was reported in the majority of cases (n= 11 ,84.6%) with stone(s) being found most frequently in the Ureter (n=6) followed by the Kidney (n=3) and both in two cases. (Fig.1) Two cases did not have any radiological evidence of urolithiasis but had stricture at the Pelvic-ureteric junction (PUJ) in affected kidney. Renal scintigraphy showed that the affected kidney’s function ranged from 0% to 12%. A pre-operative Percutaneous Nephrostomy Tube (PCN) was placed in 8 patients (61.5%) for drainage and decompression of the infected collecting system.

Laparoscopic surgery was performed successfully in all 13 patients with no conversion to the open approach in any of the cases. The mean operative time was 193.85 ± 48.95 minutes (range: 125-272), with intra-operative blood loss ranging from minimal to 300 mls (mean 103.8 +/- 88 mls). Only 2 (15.4%) patients required blood transfusions post-surgery. Hilar ligation was performed primarily with endo-vascular staplers (n=9, 69.2%), while Haem-O-lock clips were used in 4 cases (30.8%). A drain was placed in all patients (100%). The mean length of hospital stay was 3.08 ± 1.22 days (range: 2-6), and majority (77%) of the patients were discharged in ≤ 3 days, indicating that most cases required only brief hospitalization. Post-operative pain was not a common complaint with a median pain score of three at six hours on Visual analogue scale and two at 24 hours post-operatively.

Post-operative complications were seen in two (15.4%) patients: One of these patients developed urosepsis with progressively rising total leukocyte count post operatively and required change of antibiotics. The other patient experienced bradycardia and acute kidney injury with low urine output post-operatively and required observation in high dependency unit. Both complications were categorized as Clavien-Dindo Grade-II. None of our patients required readmission within 30 days. At a minimal follow-up of one year, none of the patients had developed a port site hernia and all patients were alive to date. Table-I summarizes the key findings of our cohort.

**Table-I:** Demographics, clinical, laboratory, radiological and operative details of cohort of LN for XGPN.

S. no.	Age	Gender	BMI (kg/m^2^)	Comorbids	Presenting complaints	Urine c/s	PCN tube	MAG-3	Stone location	Additional comments	Duration of surgery (min)	Estimated blood loss (mL)	length of stay (days)	Morbidity
1	56	Male	19.1	None	Flank pain, fever	no growth	Yes	02%	Ureter	Large stone in Right proximal Ureter	125	10	3	None
2	30	Female	17.3	Hepatitis C	Flank pain, fever	no growth	No	00%	Renal	Multiple obstructing calculi in Left kidney	240	50	4	None
3	77	Male	22.0	HTN, DM	Flank pain, fever, dysuria, freuqency	E. coli	Yes	12%	Both	12 mm in Left ureter, multiple calculi in Left kidney	195	50	2	None
4	26	Female	26.2	None	Flank pain	no growth	No	06%	Both	12 mm calculus in Right kidney, multiple staghorn calculi in Right kidney, 5 mm at Right distal ureter	201	100	2	None
5	57	Female	30.0	HTN, DM, IHD, CKD	Flank pain, dysuria	Pseudomonas aeruginosa	Yes	09%	Ureter	27 mm calculus in Left proximal ureter	129	50	2	None
6	37	Male	24.2	None	Flank pain	no growth	No	09%	None	Stricture at Left PUJ	144	50	3	None
7	20	Female	19.3	None	Flank pain, fever	no growth	Yes	00%	Ureter	31mm Right PUJ obstructing calculus	161	200	3	None
8	46	Male	23.7	Depression	Flan pain, fever	no growth	Yes	00%	None	Pyonephrosis seen in Left kidney	272	300	2	None
9	58	Female	19.2	Breast Ca	Flank pain, nausea	E. coli	Yes	00%	Ureter	9 mm stone in proximal ureter	268	20	5	Rising TLC postoperatively, requiring Infectious diseases (ID) review and change of antibiotics (MCG-2)
10	67	Male	21.1	Prostate Ca	Flank pain	no growth	No	00%	Renal	Multiple obstructing Right renal calculi, largest 26 mm, stricture in proximal Right ureter	183	100	6	Bradycardia, Acute kidney injury with low urine output. Managed with observation in HDU, holter monitoring, IV fluids & Antibiotic dose adjustment (MCG-2)
11	45	Female	29.1	None	Flank pain	no growth	Yes	00%	Ureter	6 mm Left mid-ureter	240	200	3	None
12	60	Female	18.9	None	Flank pain	Proteus mirabilis	No	00%	Renal	56 mm Staghorn calculi in Right kidney	162	20	2	None
13	66	Female	28.7	HTN, DM, IHD, CKD	Flank pain, dysuria, frequency	Enterococcus species	Yes	00%	Ureter	11 mm in Right distal ureter	200	200	3	None

## DISCUSSION

**Fig.2:**
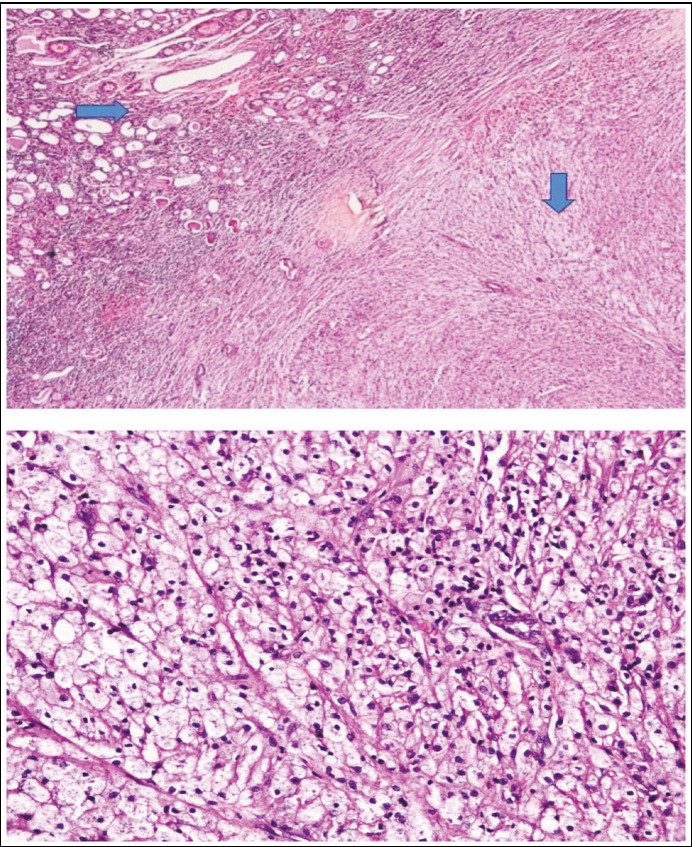
Photomicrograph of the one of the laparoscopic nephrectomy specimens exhibiting renal parenchyma (Fig2a →), partly replaced by sheets of macrophages (Fig 2a↓). High power image (40X, Fig 2b) depicts polygonal macrophages with abundant foamy cytoplasm due to deposition of lipids (xanthoma cells). Admixed lymphocytes are also seen. These macrophages are positive of CD68 (not shown here). These findings are consistent with Xanthogranulomatous Pyelonephritis (H&E, 10X & 40X).

Our study represents the first report from Pakistan investigating the surgical outcomes of Laparoscopic Nephrectomy for patients with a diagnosis of Xanthogranulomatous Pyelonephritis, a rare, severe inflammatory condition of the kidney that has proven to be a surgical challenge for urologists all over the world. 7 Xanthogranulomatous pyelonephritis (XGPN) was first described by Schlagenhaufer in 1916 as “Staphylomycosis”.^8^ It closely mimics renal cell carcinoma clinically, macroscopically and microscopically.^9^ It is marked by progressive destruction of the renal parenchyma, replaced by granulomas, abscesses, and clusters of lipid-laden macrophages (Fig.2).^10^ The inflammation has zonal pattern with bacteria, inflammatory cells seen in the inner most zone, surrounded by lipid laden macrophages. The outermost zone shows giant cells, cholesterol clefts and fibrosis with lymph aggregates.^9^

It accounts for roughly 0.6–1% of all pyelonephritis cases worldwide & most commonly affects patients in their 50s and 60s, with a female-to-male ratio of about 3:2.^10^ The population incidence is 1.4 cases per 100 000 per annum.^11^ The disease typically causes extensive inflammatory destruction of the renal parenchyma, presenting in either a diffuse form (about 83–92%), or a less frequent focal form (8–17%).^12^

The exact cause of XGPN remains unclear, but it is strongly linked to urinary tract obstruction, chronic infection, and renal stones often in patients with additional risk factors such as DM and immunocompromised status. 13 It is considered to be a potentially life-threatening condition. *Escherichia coli* and *Proteus mirabilis* account for the majority of positive cultures while *Enterococcus faecalis*, *Pseudomonas*, *Klebsiella*, *Staphylococcus aureus*, Group-B streptococci and various anaerobes represent other organisms.^8^,^12^

Standard management includes antibiotics, pre-operative decompression in majority of cases and definitive surgical treatment in form of nephrectomy.^10^ XGPN causes dense inflammation and fibrosis around the renal pelvis, hilum, and surrounding tissues, leading to distortion of normal surgical planes.^14^ Historically, an open approach has been favored as the kidney dissection is technically challenging, and laparoscopic surgery was considered contraindicated in XGPN due to these challenges with reported conversion rates of 16–33% and complication rates of 20–50%.^12^ However with advancement in surgical expertise, instrumentation, and technique, it has increasingly become a feasible option in recent times. While laparoscopic surgery involves high-cost equipment, its cost-effectiveness depends on successfully completing the procedure without complications or need for re-intervention.^15^ The benefits include reduced blood loss, less postoperative pain, smaller scars, faster recovery, shorter hospitalization and early return to work thus ultimately providing financial savings for the healthcare system.^16^

Nawaz et al. in one of the largest series from Pakistan, retrospectively reviewed 63 patients with xanthogranulomatous pyelonephritis who underwent open nephrectomy. They concluded that XGPN is common in this region, late referral often results in kidney loss, and preoperative diagnosis is crucial. When surgery is required, complete removal of all infected tissue is essential.^17^

Our cohort of 13 patients, though somewhat limited in size provides insight into patient demographics, clinical presentation and post operative outcomes specific to this population. The mean age in our cohort was 49.6 years with a clear female predominance which falls in line with what the literature has previously reported.^8^,^10^

XGPN normally presents with a wide range of possible symptoms from flank pain, fever and lower urinary tract symptoms to weight loss, nausea and gross hematuria. Among patients in our cohort flank pain was universal while systemic features such as fever and lower urinary tract symptoms were also present, consistent with the heterogeneous presentation noted in prior literature.^8^,^12^

Most of our patients (61.5%) had negative preoperative urine cultures, echoing the observation that XGPN can present without positive cultures despite significant infection and inflammation. This aligns with the largest systematic review to date, where only 59% of 1,139 patients showed positive cultures.^10^

Majority of our patients underwent preoperative drainage via PCN tube which is also consistent with the findings of contemporary literature.^10^,^16^ Most of our patients showed severely reduced kidney function on renal scans, likely due to long-standing obstruction and reluctancy of patients to seek medical care possibly due to financial constraints. This mirrors findings from a Brazilian study, where average renal function was only 8%.^18^

Our surgical outcomes affirm the feasibility and safety of laparoscopic nephrectomy for XGPN. The 100% success rate with no conversions to open surgery compares favorably with reported literature where conversion rates can range from 10% to 30% due to inflammation and adhesions.^16^,^18^,^19^ Hand-assisted techniques are described to reduce conversion rates, but this was not used in our series.

Lima et al. showed that factors including the time required to control the renal vessels, renal length and right-sided nephrectomy were associated with higher chances of conversion into an open procedure.^18^ The mean operative time in our patients (193 ± 49 minutes) was shorter than that reported by Khaira et al.^20^ (279 ± 73 minutes) and by Vanderbrink et al.^21^ (301 ±106 minutes). The low intraoperative blood loss (median 50 ml) reflects effective vessel control, particularly at the hilum. In most patients, the renal artery and vein were ligated en-bloc using an endovascular stapler, which minimized dissection time through the dense hilar adhesions. Postoperative recovery was generally uneventful, with a brief average hospital stay (~3 days) and low postoperative morbidity (15.4%), comparable to other series of laparoscopic nephrectomy for XGPN.^7^

### Limitations:

It includes its retrospective design and small sample size, which is expected to be in rare condition like XGPN. However, it provides an important starting point for understanding laparoscopic outcomes in Pakistan, where minimally invasive surgery is still evolving and many centers continue to rely on traditional open techniques. These findings can help guide current practice and support future prospective research.

## CONCLUSION

Laparoscopic nephrectomy appears to be a safe and effective approach for the management of XGPN in Pakistani patients, with favorable outcomes. Further studies are needed to see if this approach lowers complications and improves outcomes, including shorter hospital stays and early return to work, which is especially important consideration in low- and middle-income countries.

### Authors’ Contribution:

**SMN & IKJ:** Conceived, designed, did analysis & editing of manuscript.

**AAF:** Data collection and manuscript writing.

**SMN & ZU:** Manuscript writing & review, critical analysis and drafting of table and figures.

**SMN:** Takes the responsibility and is accountable for all aspects of the work in ensuring that questions related to the accuracy or integrity of any part of the work are appropriately investigated and resolved.
